# Oral and intranasal vaccines against SARS‐CoV‐2: Current progress, prospects, advantages, and challenges

**DOI:** 10.1002/iid3.604

**Published:** 2022-03-10

**Authors:** Sanchita Kar, Popy Devnath, Talha B. Emran, Trina E. Tallei, Saikat Mitra, Kuldeep Dhama

**Affiliations:** ^1^ Department of Infectious Disease Institute of Developing Science and Health Initiatives, ECB Chattar Dhaka Bangladesh; ^2^ Department of Microbiology University of Chittagong Chittagong Bangladesh; ^3^ Department of Microbiology Noakhali Science and Technology University Noakhali Bangladesh; ^4^ Department of Pharmacy BGC Trust University Bangladesh Chittagong Bangladesh; ^5^ Department of Biology, Faculty of Mathematics and Natural Sciences Sam Ratulangi University Manado North Sulawesi Indonesia; ^6^ Division of Sustainable Use of Wallacea Area The University Centre of Excellence for Biotechnology and Conservation of Wallacea, Institute for Research and Community Services, Sam Ratulangi University Manado North Sulawesi Indonesia; ^7^ Department of Pharmacy, Faculty of Pharmacy University of Dhaka Dhaka Bangladesh; ^8^ Division of Pathology ICAR‐Indian Veterinary Research Institute, Izatnagar Bareilly Uttar Pradesh India

**Keywords:** COVID‐19, intranasal vaccines, mass vaccination, mucosal vaccines, oral vaccines, SARS‐CoV‐2

## Abstract

**Background:**

The severe acute respiratory syndrome coronavirus 2 (SARS‐CoV‐2) has caused a deadly pandemic in the 21st century, resulting in many deaths, economic loss, and international immobility. Vaccination represents the only mechanism to defeat this virus. Several intramuscular vaccines have been approved and are currently used worldwide.

**Main body:**

However, global mass vaccination has not been achieved owing to several limitations, including the need for expertise to administer the injection‐based vaccine, improper distribution of the vaccine, and lack of cold chain facilities, particularly in resource‐poor, low‐income countries. Mucosal vaccines are typically administered either orally or nasally, and several studies have shown promising results for developing these vaccines against SARS‐CoV‐2 that might serve as viable alternatives to current vaccines. SARS‐CoV‐2 invades the human body via oral and nasal mucosal surfaces; thus, an oral or nasal vaccine can trigger the immune system to inhibit the virus at the mucosal level, preventing further transmission via a strong mucosal and systematic immune response. Although several approaches toward developing a mucosal vaccine are currently being tested, additional attention is required.

**Conclusion:**

In this article, the current approaches used to develop effective oral and nasal mucosal vaccines against SARS‐CoV‐2 and their benefits, prospects, and challenges have been summarized.

## Graphical abstract

Mucosal vaccines are typically administered either orally or nasally, and several studies have shown promising results for developing these vaccines against severe acute respiratory syndrome coronavirus 2 (SARS‐CoV‐2) that might serve as a viable alternative to current vaccines. SARS‐CoV‐2 invades the human body via oral and nasal mucosal surfaces; thus, an oral or nasal vaccine can trigger the immune system to inhibit the virus at the mucosal level, preventing further transmission via a strong mucosal and systematic immune response.

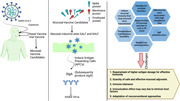

## 1 INTRODUCTION

In December 2019, a new respiratory disease, named coronavirus disease 2019 (COVID‐19), was first reported in Wuhan, China. This disease is caused by a positive‐stranded respiratory RNA virus identified as severe acute respiratory syndrome coronavirus 2 (SARS‐CoV‐2). Since then, COVID‐19 has spread globally across more than 220 countries.^1^, ^2^, ^3^, ^4^ To date (January, 2022), more than 298 million people have been infected with SARS‐CoV‐2, and over five million deaths have been reported worldwide.^5^ Those affected by this ongoing pandemic have flooded the healthcare systems of low‐income countries and impacted the advanced healthcare systems and national preparedness approaches of many high‐income nations.^6^

Several drugs, therapies, and immunomodulatory regimens, such as remdesivir, ivermectin, dexamethasone, convalescent plasma therapy, antibody‐based immunotherapies, and monoclonal antibodies have been applied under emergency use approvals to reduce disease severity among patients with COVID‐19; however, the efficacy of these treatments remains controversial, with no unambiguous evidence of treatment success.^7^, ^8^ Scientists internationally have focused on developing vaccines, with successful vaccine development viewed as imperative and representing the best option for combating the virus. Mass vaccination can prevent the severe health conditions associated with COVID‐19, reducing the burden on healthcare systems and minimizing economic losses.^9^

This crisis has sparked an unprecedented race to develop vaccines using both existing and novel vaccinology expertise. Historically, before any vaccine could be used in clinical trials, 10–15 years of research, development, and testing were required.^10^ However, in early 2020, scientists embarked on an unprecedented record‐breaking effort to develop safe and effective vaccines against SARS‐CoV‐2. More than 180 vaccine development projects were launched worldwide, many of which involved active preclinical trials in animals.^10^, ^11^ According to a recent World Health Organization (WHO) report, 132 vaccines are currently in Phase 1–3 clinical trials and an additional 194 are in preclinical development. In addition to conventional approaches to human vaccine development, various newly developed technologies have been applied for the first time.^10^, ^12^, ^13^ To date, at least seven vaccines (Covishield, Janssen/Ad26, Moderna COVID‐19, Sinopharm, Sinovac‐CoronaVac, Pfizer/BioNtech, and COVAXIN) have been authorized for human use against SARS‐CoV‐2, most of which are administered through intramuscular (IM) injections.^14^, ^15^, ^16^, ^17^, ^18^, ^19^

Similar to other ground‐breaking scientific technologies, vaccines against SARS‐CoV‐2 have become more widely available in high‐income countries much sooner than in low‐income countries.^20^, ^21^, ^22^ Because of widespread vaccination, the health situation in high‐income countries will likely continue to improve with a return to normal life, whereas low‐ and middle‐income countries, such as those in Latin America and South Asia, have already begun to experience waves of rising cases from vaccine scarcity. In addition, mild to severe side effects have been reported in vaccinated people, resulting in low vaccination compliance.^23^, ^24^ 

More attention is required to develop alternative vaccines that can minimize the challenges associated with the currently available IM vaccines, such as requiring injection and experienced health workers to administer the vaccines and the lack of cold chain maintenance to ensure vaccine effectiveness. Mucosal vaccines delivered through intranasal (IN) and oral routes represent a promising option to induce mucosal immunity.^25^, ^26^, ^27^ The IM vaccines induce a systemic immune response without eliciting a mucosal immune response; however, mucosal immunity is essential for neutralizing SARS‐CoV‐2 in the upper respiratory tract,^28^ preventing the viral spread into the lower respiratory tract and developing advanced disease stage. The absence of mucosal protection through producing local secretory immunoglobulin A (sIgA) antibodies increases the risk of SARS‐CoV‐2 transmission from vaccinated people who can still become infected and spread the virus.^28^

Recently, tremendous progress has been made toward developing mucosal vaccines, which can be delivered via oral or intranasal routes, are easy to administer due to the noninvasive route, and can generate mucosal immunity. Such immunity is separate from humoral and cellular immunity, rendering protection against COVID‐19.^29^, ^30^ In this context, several novel oral and intranasal vaccines against SARS‐CoV‐2 are currently being developed, with encouraging preclinical findings in nonhuman primates and other animal models.^13^, ^31^, ^32^, ^33^, ^34^, ^35^ Studies have also suggested that an IM vaccination followed by an oral or intranasal vaccination leads to a robust immune response, serving as a reliable approach to attaining herd immunity among the population.^29^, ^36^ Oral and intranasal vaccines can elicit a substantial B and T cell‐mediated immune response, together with the desired mucosal immunity. In addition to providing immunity, oral or intranasal vaccines are easy to administer outside of hospital settings, which may hasten the vaccination process.

If we overlook everything else, we need to vaccinate people against emerging variants of concern through effective boosters that are expected to maintain or boost immunity in the face of newer variants' emergence. The virus is continually changing and causing successive waves. According to the WHO, there are already five variants^37^ of concerns with considerable amino acid substitution, and omicron is currently spreading around the world,^38^ including Bangladesh, where approximately 32%^39^ of the population receiving two doses of vaccination. The vaccine efficacy against the newer variants of concern is always in face of threats.^37^ So, it is high time to introduce an alternative vaccination route that is easily administered for multiple boosters.

Although the world is anticipating a return to pre‐pandemic life following vaccination, children aged 12–16 years can be vaccinated now, with younger children to follow over the next few months. Multiple studies on COVID‐19 in children have reported lower infection rates, fewer symptoms, and lower mortality than in adults. However, young children with comorbidities have a higher risk of developing severe COVID‐19 than healthy children, accompanied by a high mortality rate.^40^, ^41^ Besides the direct health risks associated with COVID‐19, the indirect effects are equally concerning, as they have confined children to a deskbound lifestyle, making them idle and physically inactive, and therefore increasing the obesity rate among children. Other chronic diseases, such as diabetes, cardiovascular disease, and some cancers are gradually becoming more prominent.^42^, ^43^, ^44^ Children are the silent victims of this pandemic^45^, ^46^, ^47^; therefore, they should also be included in vaccination programs in almost all countries. Currently, IM vaccines are authorized for persons 18 years and older, although the Pfizer vaccine was recently authorized for use in children 12 years and older.^48^ Bangladesh government has also started administration of Pfizer vaccination to school‐going children aged 12–17 years.^49^ Oral or intranasal vaccination routes are often the most effective and convenient methods to vaccinate children as well as block transmission.^50^ Although several approaches are being investigated, further research is required before such vaccines can be administered to children.^51^

This article provides an overview of the potential successes and challenges faced in developing an effective mucosal vaccine that can be administered through oral and nasal routes and current progress in the design and development of vaccines to defeat SARS‐CoV‐2.

## 2 MECHANISM OF MUCOSAL VACCINES IN INDUCING IMMUNITY

The mucosal system is a common entry point for pathogens entering the body and is the first line of defense against infection. The mucosal immune system can be divided into two parts: immune‐inducing and immune effector sites. The two most important mucosal immunity inductive sites are gut‐ and nasopharynx‐associated lymphoid tissues.^52^ Oral and intranasal vaccines activate the immune system when they contact these mucosal inductive sites. Mucosal inductive sites are covered with follicle‐associated epithelia, primarily comprising microfold cells that transport foreign material (antigens) to antigen‐presenting cells (APCs).^53^, ^54^ The APCs activate effector T cells, producing cytokines that stimulate follicular plasma B cells to produce IgA antibodies. The IgA‐producing B cells travel to effector sites through systemic circulation, where they release sIgA antibodies.^55^ The sIgA antibodies are transported across the mucosal surface via a polymeric IgA receptor, where they inhibit pathogen entry through immune exclusion, antigen excretion, and intracellular neutralization.^56^, ^57^ Serum‐derived IgA and IgG antibody responses have also been documented in humans after mucosal vaccination, which protects the host cell from pathogens by performing a diverse range of effector functions.^58^

## 3 DEVELOPMENT OF POTENTIAL MUCOSAL VACCINES AGAINST SARS‐COV‐2

Vaccination is the most effective method for combating the COVID‐19 pandemic. All current WHO‐approved vaccines against SARS‐CoV‐2 are administered via the parenteral IM route,^59^ inducing high systemic neutralizing antibody titers that can neutralize systemic viral infections; however, they are ineffective in producing efficient mucosal immunity.^60^ Mucosal vaccines provide a new avenue for combating SARS‐CoV‐2 because they can generate efficient immune responses at the mucosal and systemic levels.^61^ Mucosal vaccines can be delivered orally, intranasally, rectally, vaginally, ocularly, or sublingually, although oral and intranasal delivery routes are the most commonly used. Oral immunization induces strong immune responses in the gastrointestinal tract and mammary and salivary glands, whereas intranasal immunization provides noticeable antigen‐specific immune responses in the respiratory, gastrointestinal, and genital tracts. Mucosal‐based immunizations, such as oral polio,^62^ rotavirus,^63^, ^64^ typhoid fever,^65^ and intranasal spray of influenza^66^, ^67^ vaccines are alternative approaches to induce preventive immunity against various enteric and respiratory infectious diseases.

SARS‐CoV‐2 primarily enters the host system via mucosal routes and is then transmitted through the mucosal membranes of the eyes, nose, and mouth. The virus enters the respiratory tract through mucosal barriers and invades the underlying mucosal and epithelial layers (lungs).^68^ Studies on the SARS‐CoV‐2 spike (S) protein have revealed that it recognizes the human angiotensin‐converting enzyme 2 receptor, which represents the first point of entry into the host cell.^69^, ^70^, ^71^ The angiotensin‐converting enzyme 2 receptors recognized by SARS‐CoV‐2 is highly expressed on cell surfaces that comprise the mucosal linings of the oral and nasal epithelia and on enterocytes of the digestive system, such as the ileum and colon.^72^, ^73^ The expression level of the angiotensin‐converting enzyme 2 receptor is low in the alveoli^74^ and viral replication is higher in the oral and nasal sites than in the alveoli.^75^

Understanding the roles played by the nasal and gastric mucosa in SARS‐CoV‐2 transmission and disease progression helps support the potential benefits of mucosal immunization using oral or intranasal vaccines.^30^ Mucosal vaccines have successfully been used previously to prevent infectious diseases affecting the gastrointestinal and respiratory tracts, and they have been effective in inducing and activating the mucosal immune system.^76^ For example, the live oral enteric‐coated adenovirus types 4 and 7 vaccines are approved for use in the US military personnel aged 17–50 years and are safe and highly effective in reducing disease burden and inducing mucosal immunity against adenovirus‐associated respiratory illnesses.^77^ An orally administered influenza vaccine recently completed Phase II clinical trials and generated protective immunity compared with the immunity generated by a licensed IM vaccine.^78^ These findings represent significant progress in developing oral vaccines for respiratory diseases and provide confidence for the eventual successful development of mucosal vaccines against SARS‐CoV‐2 that can be administered through oral or intranasal routes. We reviewed the oral and intranasal COVID‐19 vaccine candidates that are currently in the development pipeline or have entered the preliminary clinical stages and these are summarized in Table 1.

**Table 1:** Mucosal COVID‐19 vaccine candidates in clinical and preclinical development

Sl no.	Vaccine platform	Vaccine candidates	Vaccine target	Developer	Clinical stage	No of doses	Schedules (days)	Route of administration	References
1.	Adenoviral vector (nonreplicating)	VXA‐CoV2‐1	Full‐length spike (S) protein and the nucleocapsid (N) proteins of SARS‐CoV‐2	Vaxart	Preclinical trial completed	2	0 + 28	Oral	Ashraf et al.^12^; Kyriakidis et al.^13^; Kaur and Gupta^79^; Rawat et al.^80^; Moore et al.^81^
2.	Adenoviral vector (nonreplicating)	ChAdOx1‐S ‐ (AZD1222)(Covishield)	SARS‐CoV‐2 S protein	University of Oxford	Phase 1: NCT04816019	1–2	0 + 28	IN	ClinicalTrials.gov ^82^
3.	Viral vector (nonreplicating)	OraPro‐COVID‐19™	SARS‐CoV‐2 S protein	isoBio, UK (previously known as Stabilitech), in collaboration with BioCell Corporation (New Zealand)	Not Available	Not Available	Not Available	Oral	Ashraf et al.^12^; Stabilitech^83^, ^84^
4.	Adenoviral vector (nonreplicating)	Human Adenovirus type 5: hAd5 S + N vaccine (S‐fusion + N‐ETSD) E2b‐deleted Adeno	Pre‐fusion, stabilized SARS‐CoV2 S protein	Immunity Bio. Inc	Phase 1: NCT04591717 NCT04710303	1–2 doses Day	0 + 21	Subcutaneous or Oral	Pilicheva and Boyuklieva.^85^
5.	DNA vaccine platform	BacTRL‐Spike vaccine	SARS‐CoV‐2 S protein	Canada‐based company Symvivo	Phase 1: NCT04334980	1	0	Oral	Kyriakidis et al.^13^; Silveira et al.^86^; Sharma et al.^87^
6.	Virus‐like particle (VLP)	Oravax	Three SARS‐CoV‐2 structural proteins, including the S protein, the membrane protein (M), and the small, membrane‐associated envelope protein (E).	Oramed Pharmaceuticals & Premas Biotech.	Pilot animal study completed first‐in‐human clinical trials will be started in 2021	1	0	Oral	Oramed^88^
7.	Live attenuated virus	COVI‐VAC	Segment of SARS‐CoV2 S protein	Serum Institute of India in collaboration with Codagenix (United States	Phase 1: NCT04619628	1–2 doses	0 or 0 + 2	IN	COVI‐VAC^89^; Velikova and Georgiev.^90^
8.	Adenoviral vector (Non‐replicating)	BBV154	Stabilized spike (S) protein of SARS‐CoV‐2	Bharat Biotech	Phase 1: NCT04751682	1	0	**IN**	Sharun and Dhama.^91^
9.	Live attenuated virus	MV‐014‐212	SARS‐CoV‐2 spike (S) protein	Meissa Vaccines, Incorporated	Phase 1: NCT04798001	1	0	IN	Pilicheva and Boyuklieva.^85^
10.	Attenuated parainfluenza virus (PIV5) vector	CVXGA1	SARS‐CoV‐2 spike (S) protein	CyanVac and Blue Lake Biotechnology	Phase I trial NCT04954287	1	0	IN	Pilicheva and Boyuklieva.^85^
11.	Viral vector (replicating)	DelNS1‐2019‐nCoV‐RBD‐OPT1(Intranasal flu‐based‐RBD	Receptor‐binding domain (RBD) of spike (S) glycoprotein SARS‐CoV‐2	University of Hong Kong, Xiamen University and Beijing Wantai Biological Pharmacy	Phase 1:ChiCTR2000037782NCT04809389Phase 2:ChiCTR2000039715	2 doses	0 + 28	IN	Martínez‐Flores et al.^92^
12.	RNA‐based vaccine	LNP‐nCoVsaRNA	Spike (S) protein	Imperial College London	Phase 1: ISRCTN17072692	2	0 + 28	IN	McKay et al.^93^
13.	RNA‐based vaccine	SARS‐CoV‐2 mRNA vaccine (ARCoV)	RBD of S protein	Academy of Military Science (AMS), Walvax Biotechnology and Suzhou Abogen Biosciences	Phase 1: ChiCTR2000034112 ChiCTR2000039212 Phase 2: ChiCTR2100041855	2	0 + 28	IN	Zhang et al.^94^; Yan et al.^95^
14.	Protein subunit	CoV2‐OGEN1, protein‐based vaccine	RBD of S protein	USSF/Vaxform	Phase 1: NCT04893512	1–2	Day 0 + /− 14	Oral	CoV2‐OGEN1^96^
15.	Bacterial antigen‐spore expression vector	COVID‐19 oral vaccine consisting of *Bacillus subtilis* spores	Spike (S) protein	DreamTec Research Limited	Not available	3	Day 0 + 14 + 28	Oral	COVID‐19 oral vaccine^97^
16.	Protein subunit	CIGB‐669 (RBD + AgnHB)	RBD of S protein	Center for Genetic Engineering and Biotechnology (CIGB)	Phase 1/2: RPCEC00000345	3	0 + 14 + 28 or 0 + 28 + 56	IN	Jia and Gong.^98^
17.	Protein subunit	CIGB‐66 (RBD + aluminum hydroxide)	RBD of S protein	Center for Genetic Engineering and Biotechnology (CIGB)	Phase 1/2: RPCEC00000346 Phase 3 RPCEC00000359	3 doses	0 + 14 + 28 or 0 + 28 + 56	IN	Jia and Gong.^98^
18.	Protein subunit	Razi Cov Pars, recombinant spike protein	RBD of S protein	Razi Vaccine and Serum Research Institute	Phase 1: IRCT20201214049709N1	3	0 + 21 + 51	IM and IN	Trials IRoC^99^

Abbreviations: COVID‐19, coronavirus disease 2019; SARS‐CoV‐2, severe acute respiratory syndrome coronavirus 2.

Other new optimistic approaches have also been reported. One of these is to use different administration methods in subsequent vaccination doses, with both IM and intranasal routes. Su et al. used this approach and analyzed the resultant immune responses.^33^ In their study, two groups of rhesus macaques were immunized with subunit vaccines combined with adjuvant. One group received IM‐priming and IN booster vaccines, whereas the other group received IM‐priming and intranasal booster vaccines. Both groups showed efficient immune responses against SARS‐CoV‐2 infection, and the intranasal vaccine was effective after multiple doses (three booster doses), rapidly removing the virus from the nasal cavity and preventing viral transmission. Therefore, a complementary booster dose for conventional systemic vaccines might provide added protection.^33^

The IM‐priming and IN booster strategy using lentiviral vector‐based vaccines produce neutralizing antibodies against the SARS‐CoV‐2 S protein. This vaccine candidate has been tested in two animal models: mice and golden hamsters.^34^ The IM immunization of mice using this vaccine elicited a high level of neutralizing antibodies; however, it only provided partial protection. Subsequent administration of an intranasal booster dose showed a significant decrease in lung viral loads and reduced local inflammation. In the golden hamster animal model, which closely mimics the COVID‐19 physiopathology observed in humans, intranasal immunization with this vaccine platform showed a significant prophylactic effect preventing excessive lung injury.^34^ Another intranasal subunit vaccine containing trimeric or monomeric S protein combined with adjuvant (liposomal stimulator of interferon genes agonist) induced strong mucosal and systemic immunity in mice.^100^

Another study researched generating a cold‐adapted, live, attenuated vaccine by adjusting SARS‐CoV‐2 growth in Vero cells at temperatures of 37°C to 22°C. A single intranasal dose of the SARS‐CoV‐2/human/Korea/CNUHV03‐CA22°C/2020 vaccine delivered to mice elicited a significant B and T cell‐mediated immune response and induced mucosal IgA antibodies, fully protecting the animals against infection from SARS‐CoV‐2.^101^ Therefore, nasal vaccination could represent a viable mechanism for eliciting a strong immune response.^12^, ^101^ The SARS‐CoV‐2 N protein has also received attention as a potential vaccine target. The intranasal vaccination of BALB/c mice with a recombinant Adenovirus type 5 (Ad5) expressing the SARS‐CoV‐2 N protein‐induced significant quantities of CD8^+^ and CD4^+^ T cell‐mediated immune responses, whereas intradermal administration induced a less robust response. The CD4^+^ T cell‐mediated immune response has been associated with an enhanced antibody‐mediated immune response.^102^

Several studies have proposed the design of molecular vaccines combined with potential adjuvants based on nanotechnology advancements. Using nanoparticles as an adjuvant allows these vaccines to be administered intranasally and significantly induces systemic and mucosal immunity.^103^, ^104^, ^105^

## 4 ADVANTAGES OF ORAL AND INTRANASAL VACCINES AGAINST SARS‐COV‐2 OVER INTRAMUSCULAR VACCINES

### 4.1 Wide immune response

All currently approved SARS‐CoV‐2 vaccines are administered via the IM route, which primarily induces immunity in the peripheral and lower respiratory tracts^29^ but not in the upper respiratory tract, whereas mucosal immunity serves as the first line of defense against respiratory pathogens.^106^ Mucosal immunization via oral or nasal routes can effectively induce mucosal immune responses by recruiting antibodies and T cells to the wet and open surfaces where most respiratory pathogens first invade. Mucosal IgA plays a key role in viral neutralization and blocks further viral transmission.^36^ Successfully developing oral or nasal vaccines against SARS‐CoV‐2 will stop mucosal SARS‐CoV‐2 transmission and remove major barriers to global vaccine distribution and deployment, especially for low‐income countries.

### 4.2 Feasibility

A major impediment to widespread, global vaccination is the lack of cold chain infrastructure and the technology required for vaccine storage, distribution, and transportation, particularly in rural areas.^107^, ^108^ The majority of currently approved COVID‐19 vaccines require transportation and storage at low temperatures. For example, the Oxford–AstraZeneca and Sinovac COVID‐19 vaccines must be maintained at 2–8°C, and mRNA vaccines such as the Moderna COVID‐19 vaccine require a storage temperature of approximately −58°C.^109^ Strict regulations for temperature and care are crucial for maintaining vaccine efficacy, potency, and stability.^13^ The complexity involved in temperature maintenance might cause reduced immunization coverage in remote areas, increasing the probability of COVID‐19 outbreak infections. Most oral and nasal vaccine candidates under development do not require cold storage as they are designed to be heat stable and resistant to the acidic environments of the gut.^26^, ^76^

### 4.3 Simplified administration

The administration of oral or nasal vaccines does not require the presence of trained healthcare professionals because it is needle‐free in contrast to the IM vaccination process. Needle‐free administration reduces the need for trained personnel to administer vaccinations. The advantages of oral and nasal COVID‐19 vaccines might significantly extend the practical options for vaccine distribution, particularly in resource‐limited settings where other preventive measures, such as social distancing, may be more difficult to maintain.

### 4.4 Expanded compliance

The goal of any vaccination campaign is to protect people from infectious diseases by developing a sufficient level of herd immunity, preventing further viral transmission. Experts support the attainment of herd immunity to SARS‐CoV‐2 through natural infection or vaccination.^110^ Mucosal vaccines represent a promising alternative to IM vaccines because mucosal vaccine compliance is high and effective for mass immunization.^111^ Moreover, mucosal vaccines represent a better option for infants because the administration does not require injection. Recently, CDC recommends the COVID‐19 vaccine (Pfizer‐BioNTech) for children in the age of five and older. Similar to adults, children can experience side effects in response to COVID‐19 vaccines, such as sore arms, muscle aches, fever, and chills. However, oral and intranasal vaccination routes are commonly associated with reduced side effects compared to IM vaccines,^112^ which might enable children to be included in extensive COVID‐19 vaccination programs.

## 5 CHALLENGES TO THE DEVELOPMENT OF COVID‐19 MUCOSAL VACCINES THAT CAN BE ADMINISTERED INTRANASALLY AND ORALLY

### 5.1 Ineffective long‐lasting immunity

Conventionally, mucosal vaccines administered orally or intranasally require higher doses than those administered via IM parenteral routes^26^ because vaccines administered via nasal or oral routes become diluted by mucus in the nasal or oral cavity and by the ciliary movement of the respiratory tract. Intranasally delivered antigens must reach the mucosal sites, cross the mucus layer, and generate local IgA production, whereas orally delivered antigens must remain intact in the low pH environment of the upper gastrointestinal tract and withstand various nucleases and proteases found in the digestive tract to successfully induce immunity.^2^

### 5.2 Scarcity of appropriate mucosal adjuvants

To overcome these obstacles, vaccines are often complemented with an adjuvant (chitosan, poly lactic‐co‐glycolic acid, enteric‐coated gelatin capsules, or the inclusion of copolymeric microparticle liposomes or proteasomes) when delivered via oral or nasal routes.^113^, ^114^, ^115^, ^116^, ^117^ Limited options are currently available for use as adjuvants for administering human mucosal vaccines, and large quantities of adjuvant are required because microparticles become trapped in the mucus, resulting in only a small fraction of the administered vaccine achieving entry to the mucosal immunological sites.^25^ The use of live, attenuated virus or live viral vectors in intranasal immunizations is associated with a risk of harmful antigens and adjuvants (such as cholera toxin and *Escherichia coli* enterotoxin) gaining entry to the central nervous system through the cribriform plate.^25^ These possibilities emphasize the importance of evaluating toxicological outcomes, in accordance with standard regulatory requirements, before the approval of nasal SARS‐CoV‐2 vaccines.^29^

### 5.3 Immune tolerance

Another concern is that mucosal vaccines that reach immunological sites might induce immune tolerance, which is a standard feature of the immune system at mucosal surfaces.^118^, ^119^ These challenges must be addressed effectively when developing oral or intranasal vaccines against SARS‐CoV‐2, and several strategies such as the accurate antigen dose to induce an immune response, appropriate adjuvant in vaccine formulation, and timing of vaccine delivery and intervals of multiple dosages have recently been investigated.^120^

### 5.4 Effect of intrinsic **host** factors

In addition to the potential for immunotolerance, the efficacy of mucosal vaccines is determined by various factors, such as age, the environment, host genetics, microbiome of the recipient, and the immunization regimen. Currently, the number of approved mucosal vaccines is very low. Oral rotavirus, influenza, and poliovirus vaccines are all widely used in humans. The efficacy of orally administered rotavirus vaccines ranges from 70% to 90%, and the efficacy for oral vaccines against influenza and polioviruses ranges from 85% to 90%.^61^ The underlying health status of vaccinated people can also affect efficiency, leading to disparate outcomes between high‐ and low‐income countries. For example, oral vaccines for cholera, polio, and rotavirus are less effective in low‐income countries than in high‐income countries. Several factors, including nutritional deficiencies and low levels of vitamin A or zinc; concurrent bacterial, parasitic, or viral infections; and high levels of maternal antibodies in breastmilk can reduce vaccine efficacy.^121^, ^122^, ^123^

In human challenge studies, the immunity acquired following coronavirus infections is frequently transient and, in some cases, re‐infection with the same virus is possible after an extended period.^124^ SARS‐CoV‐2 infections are particularly severe in older patients (typically those older than 50 years),^125^ and older individuals often do not respond prominently to vaccination regarding neutralizing antibody titers and require higher antigen levels to produce sufficient immunogenicity.^126^, ^127^ All these issues must be addressed during the design and development of an effective mucosal vaccine against SARS‐CoV‐2. Before extending vaccination to vulnerable age groups and immunocompromised people, the immunization effects of vaccine doses should be investigated.

## 6 CONCLUSION AND PROSPECTS

The WHO has accelerated the vaccine development process to expedite global access to safe, effective, and high‐quality vaccines against SARS‐CoV‐2. Almost all available vaccines are delivered via the IM route and have shown high levels of efficacy, eliciting a significant immune response. However, herd immunity is almost impossible to achieve using only current vaccinations due to many limitations, causing barriers to mass vaccination efforts. Vaccine distribution, accessibility, and administration should be equitable worldwide, especially in high‐risk areas and among vulnerable groups in low‐income countries. A recent study revealed that mucosal vaccines (intranasal) could be used as a booster dose after a primary dose of IM vaccination, resulting in a strong immune response preventing replication of SARS‐CoV‐2 in the upper and lower respiratory tracts.^29^ Mucosal vaccines can be used in addition to current IM vaccines in situations where IM vaccine administration is difficult.

Intense research using advanced methodology is required to develop promising mucosal vaccines with high efficacy and few adverse reactions in humans. Several approaches from previous studies and data from previously used mucosal vaccines must be investigated to develop a novel mucosal vaccine against SARS‐CoV‐2.

Developing effective and reliable mucosal vaccines by adopting nonconventional approaches is crucial. In the future, the availability of potential mucosal vaccines might become feasible after further assessment of the vaccines currently being developed, including the evaluation of large‐scale clinical studies and trials, leading to their incorporation in worldwide vaccination programs.

## CONFLICTS OF INTEREST

The authors declare no conflicts of interest.

## AUTHOR CONTRIBUTIONS

**Sanchita Kar**: Conceptualization, Data curation, Writing‐Original draft preparation, Writing‐ Reviewing and Editing. **Popy Devnath**: Data curation, Writing‐Original draft preparation, Writing‐ Reviewing and Editing. **Talha Bin Emran**: Conceptualization, Writing‐Reviewing, and Editing, Visualization. **Trina Ekawati Tallei**: Conceptualization, Supervision. **Saikat Mitra**: Data curation, Writing‐Reviewing, and Editing. **Kuldeep Dhama**: Writing‐ Reviewing and Editing, Visualization, Supervision.

## Data Availability

The data that support the findings of this study are available from the corresponding author upon reasonable request.
